# A dynamic immune response index combining C-reactive protein and lactate dehydrogenase kinetics is associated with outcomes of PD-1 inhibitor monotherapy in recurrent/metastatic nasopharyngeal carcinoma

**DOI:** 10.3389/fonc.2026.1775584

**Published:** 2026-03-13

**Authors:** Jia-min Chen, Su-chen Li, Rui-xin Liu, Zi-jun Yin, Qiang Quan, Da-feng Ling, Hao-xiang Long, Li Yuan, Meng Xu, Lin-quan Tang, Wen-hui Chen

**Affiliations:** 1 Department of Oncology, The First Affiliated Hospital of Jinan University, Guangzhou, China; 2 Department of Nasopharyngeal Carcinoma, Sun Yat-sen University Cancer Center, Guangzhou, China; 3 State Key Laboratory of Oncology in South China, Guangzhou, China; 4 Collaborative Innovation Center for Cancer Medicine, Guangzhou, China; 5 Guangdong Key Laboratory of Nasopharyngeal Carcinoma Diagnosis and Therapy, Guangzhou, China

**Keywords:** C-reactive protein, immune response index, lactate dehydrogenase, PD-1, recurrent or metastatic nasopharyngeal carcinoma

## Abstract

**Background:**

Reliable biomarkers associated with outcomes of PD-1 inhibitor monotherapy in recurrent/metastatic nasopharyngeal carcinoma (RM-NPC) are lacking. We developed an immune response index (IRI) integrating C-reactive protein (CRP) and lactate dehydrogenase (LDH) to address this need.

**Methods:**

In 209 RM-NPC patients receiving PD-1 inhibitor monotherapy, we constructed a pretreatment IRI using baseline CRP and LDH, and an on-treatment IRI based on their early kinetic changes. Associations with objective response rate (ORR), progression-free survival (PFS), and overall survival (OS) were analyzed.

**Results:**

The overall ORR was 23.4% (median PFS: 3.5 months; OS: 20.2 months). The composite pretreatment IRI outperformed individual markers, stratifying patients into low-, intermediate-, and high-risk groups with ORRs of 47.8%, 17.3%, and 15.9%, respectively (P<0.0001). Corresponding median PFS was 7.3, 2.8, and 2.0 months. The on-treatment IRI further refined prediction: the good-response group (ORR 54.7%) had a median PFS of 7.5 months and OS of 31.9 months, while the poor-response group (ORR 9.4%) had a median PFS of 2.0 months. This dynamic model identified primary refractory disease more accurately than single-marker kinetics.

**Conclusion:**

The CRP-LDH-based IRI, both at baseline and during early treatment, is a practical tool for stratifying prognosis and dynamically monitoring response to PD-1 inhibitors in RM-NPC, which supports ongoing clinical evaluation.

## Introduction

The management of recurrent or metastatic nasopharyngeal carcinoma (RM-NPC) continues to present substantial clinical difficulties, despite progress in combined treatment approaches. Anti-PD-1 therapy has established itself as a fundamental treatment component—approved globally both in first-line combination regimens and as a valuable monotherapy alternative following unsuccessful chemotherapy. However, important constraints remain. While trials such as CAPTAIN-1st (1) and JUPITER-02 (2) have shown survival benefits with PD-1 inhibitors combined with chemotherapy, response rates to PD-1 inhibitor monotherapy are generally limited, with objective response rates usually falling between 20% and 34% (3, 4). Additionally, although some evidence suggests immunotherapy may continue for up to two years, the ideal treatment duration for RM-NPC patients has not been well established.

A pressing clinical need exists for dependable, practical biomarkers that can forecast which patients will experience sustained benefits from PD-1 inhibition and help determine appropriate treatment duration. Investigations into PD-L1 expression and plasma EBV DNA have been conducted, but both have limitations. PD-L1’s predictive value in NPC has been variable, with studies including CHECKMATE-141 showing no clear connection with survival results (5). Plasma EBV DNA, while showing promise as a monitoring biomarker (6), faces challenges in widespread clinical adoption due to inconsistent testing methodologies across different centers. Therefore, easily accessible, standardized biomarkers that dynamically reflect the tumor-host interplay are urgently needed. Systemic inflammation, a key hallmark of cancer progression and immune suppression, offers a promising avenue. Inflammatory indicators are routinely measurable and capture critical host-tumor microenvironment interactions influencing immunotherapy response.

Two particularly relevant markers are C-reactive protein (CRP) and lactate dehydrogenase (LDH), both with solid scientific and clinical foundations. CRP, produced by the liver in reaction to inflammatory signals such as IL-6, serves as a reliable measure of systemic inflammation (7). Increased CRP levels correlate with poorer outlook in multiple cancers (8, 9) and have been connected to reduced effectiveness of immunotherapy in non-small cell lung cancer (10). LDH, an enzyme involved in energy metabolism that often rises in advanced tumors, is a long-recognized prognostic marker in cancer care (11). Elevated LDH before treatment regularly corresponds to diminished responses to immune checkpoint inhibitors in conditions like melanoma (12) and lung cancer (13), possibly indicating an altered metabolic state favorable to tumor growth.

However, comprehensive data on the predictive value of CRP and LDH, particularly as dynamic biomarkers, specifically for RM-NPC patients receiving PD-1 inhibitors, are lacking. Current models rely predominantly on static, pre-treatment measurements (14). Serial monitoring of these markers during therapy could yield more nuanced, real-time insights into therapeutic efficacy and emerging resistance.

Based on these considerations, we proposed that combining CRP and LDH measurements—both initially and throughout treatment—might create a more effective prediction method. We developed a combined Immune Response Index (IRI) to categorize RM-NPC patients undergoing PD-1 inhibitor monotherapy. Our research aimed to evaluate whether the pre-treatment IRI could pinpoint patients with higher likelihood of response, and whether tracking IRI during treatment could provide early indications of therapeutic benefit, ultimately supporting more tailored treatment approaches.

## Materials and methods

### Study population

This retrospective cohort study included 209 patients with recurrent or metastatic nasopharyngeal carcinoma (RM-NPC) who received single-agent anti-PD-1 therapy at Sun Yat-sen University Cancer Center (SYSUCC) in Guangzhou, China, between April 2016 and March 2022. Patients were treated with one of the following anti-PD-1 inhibitors: camrelizumab (200 mg), toripalimab (3 mg/kg), nivolumab (3 mg/kg or 240 mg), spartalizumab (400 mg), or pembrolizumab (3 mg/kg). Treatment was administered every two weeks until disease progression, unacceptable toxicity, investigator decision, patient withdrawal of consent, or for a maximum of two years.

Baseline and on-treatment laboratory data, including serum levels of C-reactive protein (CRP), lactate dehydrogenase (LDH), albumin, neutrophil-to-lymphocyte ratio (NLR) and plasma EBV-DNA, were retrieved from electronic medical records. Demographic, clinical, and pathological information were also collected. All blood biomarker assays were conducted in the hospital clinical laboratory, employing rigorous internal standardization and quality control protocols. Detailed methodological information is available in **Supplementary Table 1**. Plasma EBV-DNA was quantified using real-time quantitative polymerase chain reaction (15). As a retrospective analysis, this study was granted a waiver of informed consent by the institutional review board.

### Definition of CRP and LDH kinetics

Patients were classified based on previously established CRP kinetic criteria (16). CRP flare-response was defined as at least a twofold increase from baseline within 30 days after initiating ICI therapy, followed by a subsequent decrease in serum CRP below baseline levels. CRP response was defined as a reduction in serum CRP to below 30% of baseline at least once within the first 12 weeks of treatment. Patients who did not meet either of these criteria were classified as CRP non-responders.

LDH kinetics were categorized as follows: LDH flare-response was defined as a sustained decrease in LDH levels below baseline throughout a 12-week observation period. In contrast, LDH non-response was defined as LDH levels persistently remaining above baseline during the same 12-week period. Patients whose LDH levels fell below baseline at least once during ICI treatment, but not consistently throughout the entire period, were classified as LDH responders.

Serum CRP and LDH were measured serially on a biweekly schedule, synchronized with the 2-week PD-1 inhibitor treatment cycle. Blood samples were collected immediately prior to each scheduled infusion.

### Definition of immune response index

The pre-treatment Immune Response Index (IRI) was constructed using two baseline laboratory parameters: CRP >4.74 mg/L and LDH >240 U/L. Patients were stratified into three risk categories based on the number of elevated parameters: low risk (0 elevated factors), intermediate risk (1 elevated factor), and high risk (2 elevated factors).

The on-treatment immune response index (IRI) was derived from the combined kinetic patterns of CRP and LDH measured during therapy. Initially, for each biomarker, patients whose pattern met either the flare-response or response criteria were categorized as “responders”; all others were classified as “non-responders.” Using this combined response status, patients were stratified into three prognostic groups. The good-response group included those who were responders for both CRP and LDH. The intermediate-response group comprised patients who were responders for one biomarker only. The poor-response group consisted of patients who were non-responders for both biomarkers.

### Study endpoint

The primary endpoint of this study was objective response rate (ORR), defined as the proportion of patients achieving a complete or partial response as per RECIST version 1.1. Secondary endpoints included overall survival (OS) and progression-free survival (PFS). PFS was calculated from the initiation of immune checkpoint inhibitor (ICI) treatment to the first occurrence of disease progression, death from any cause, or the last follow-up for censored patients. OS was measured from the start of ICI therapy to death from any cause or the last follow-up for censored patients. Both disease progression and death were considered events for survival analyses.

### Statistical analysis

Categorical variables were compared using the χ^2^ test or Fisher’s exact test, as appropriate. Continuous variables were analyzed using unpaired t-tests or the Wilcoxon rank-sum test, depending on data distribution. To ensure consistency and objectivity, optimal cutoff values for CRP, LDH, albumin, NLR, and EBV-DNA were determined by receiver operating characteristic (ROC) curve analysis based on ORR (**Supplementary Figure 1**).

Survival outcomes were estimated using the Kaplan Meier method, with between-group comparisons assessed by the log-rank test. Prior to constructing the multivariable Cox model, all candidate variables were assessed for multicollinearity using variance inflation factors (VIF). Only variables with a VIF < 3 were retained for further analysis. Subsequently, variables showing a significance level of p < 0.05 in univariate analysis were selected, and a forward likelihood ratio (LR) procedure was applied to develop the most parsimonious final model. A two-sided P-value <0.05 was considered statistically significant. All statistical analyses were conducted using SPSS version 25.0 (SPSS Inc., Chicago, IL, USA) and R version 4.0.2 (R Foundation for Statistical Computing, Vienna, Austria).

## Results

### Patient population

The baseline clinicopathological characteristics of the 209 patients with RM-NPC treated with PD-1 inhibitor monotherapy are summarized in **Table 1**. In the overall cohort, the ORR was 23.4%. The median PFS was 3.5 months (95% CI, 2.0–3.8), and the median OS was 20.2 months (95% CI, 15.4–25.5). The median baseline levels of CRP and LDH were 11.98 mg/L (range, 0.17–220.18) and 240.7 U/L (range, 118.4–5321.6), respectively.

**Table 1:** Clinical and pathological characteristics of the 209 patients.

Clinical characteristic	Overall cohort n=209
Age, y	
Median (range)	47 (25-72)
≤45	92 (44.0)
>45	117 (56.0)
Sex	
Male	178 (85.2)
Female	31 (14.8)
Body mass index, kg/m^2^	
<18.5	34 (16.3)
18.5-23.9	139 (66.5)
23.9-27.9	32 (15.3)
≥28.0	4 (1.9)
EOCG	
0	66 (31.6)
1	143 (68.4)
Smoking history	
No	146 (69.9)
Yes	63 (30.1)
Drinking history	
No	193 (92.3)
Yes	16 (7.7)
Disease type	
Metastasis	164 (78.5)
Recurrence	15 (7.2)
Both	30 (14.4)
No of metastatic sites	
0	15 (7.2)
1-3	10 (4.8)
≥4	184 (88.0)
EBV-DNA, copy/ml	
≤1490	45 (21.5)
>1490	164 (78.5)
ALB, g/L	
≤44	123 (58.9)
>44	86 (41.1)
CRP, mg/L	
≤4.74	64 (30.6)
>4.74	145 (69.4)
LDH, U/L	
≤240	103 (49.3)
>240	106 (50.7)
NLR	
≤3.7	13 (6.2)
>3.7	196 (93.8)
Tumor response	
CR/PR	49 (23.4)
SD	71 (34.0)
PD	89 (42.6)

ECOG, Eastern Cooperative Oncology Group Performance Status; NPC, Nasopharyngeal carcinoma; EBV-DNA, Epstein-Barr virus DNA; ALB, Albumin; CRP, C-reaction protein; LDH, Lactate dehydrogenase; CR, Complete responses; PR, Partial response; SD, Stable disease; PD, Progressive disease.

**Figure 1:**
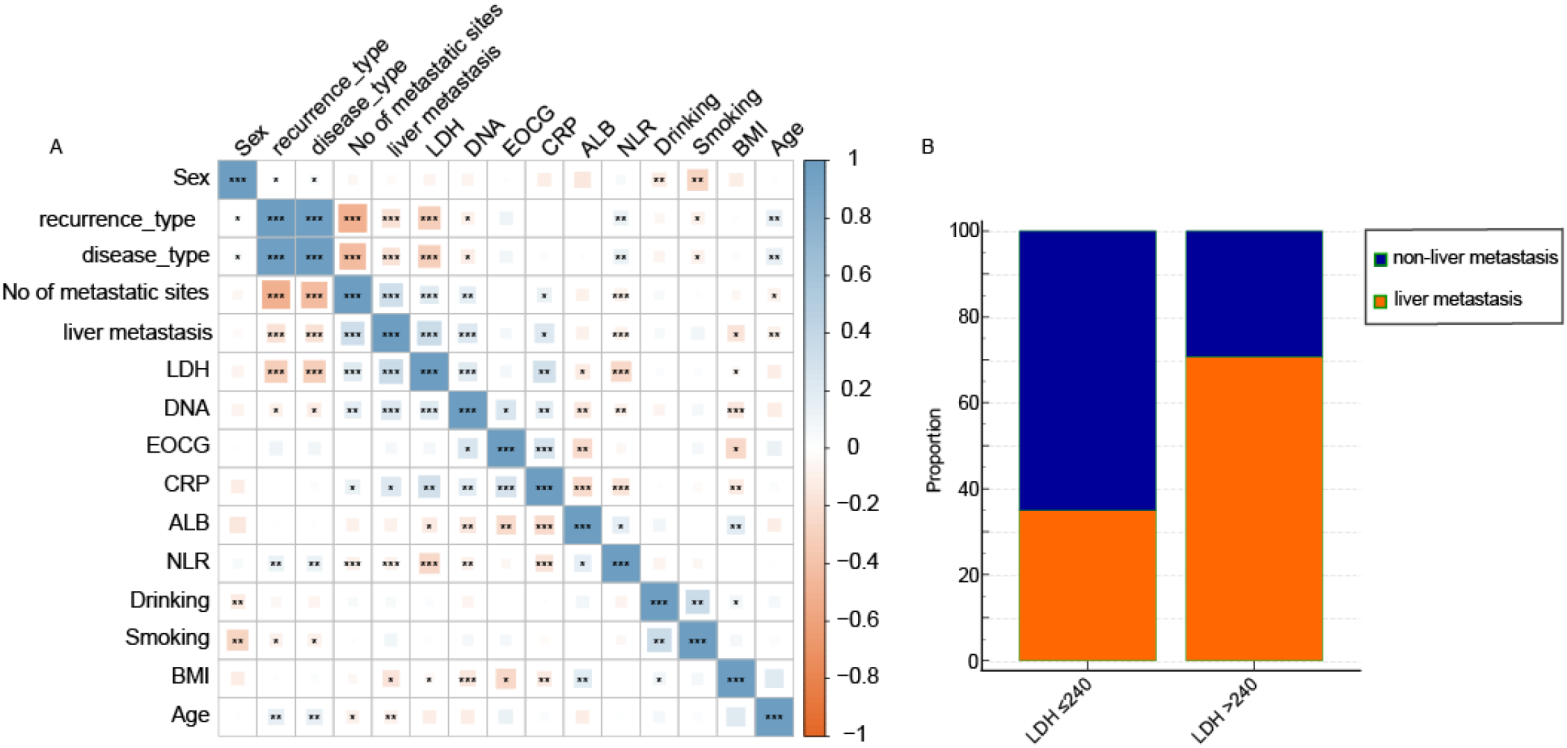
Baseline inflammatory markers and their association with clinicopathological characteristics. **(A)** Correlation heatmap of pretreatment levels of blood biomarkers with key clinical and pathological features. Significance levels are denoted as follows: *P < 0.05, **P < 0.01, ***P < 0.001. **(B)** Representative box plot illustrating the significant association between elevated baseline LDH levels and the presence of liver metastasis.

A strong association was observed between baseline LDH levels and the presence of liver metastasis (**Figure 1A**). Among patients with baseline LDH >240 U/L, 70.8% had liver metastases, compared to 35.0% of those with LDH ≤240 U/L (P < 0.001, **Figure 1B**).

A total of 186 patients had evaluable longitudinal biomarker data and were included in the subsequent dynamic analysis. The remaining 23 patients were excluded due to early disease progression, loss to follow-up, or withdrawal of consent before the first on-treatment assessment at 4 weeks.

### Association of baseline CRP and LDH with treatment outcomes

**Figure 2:**
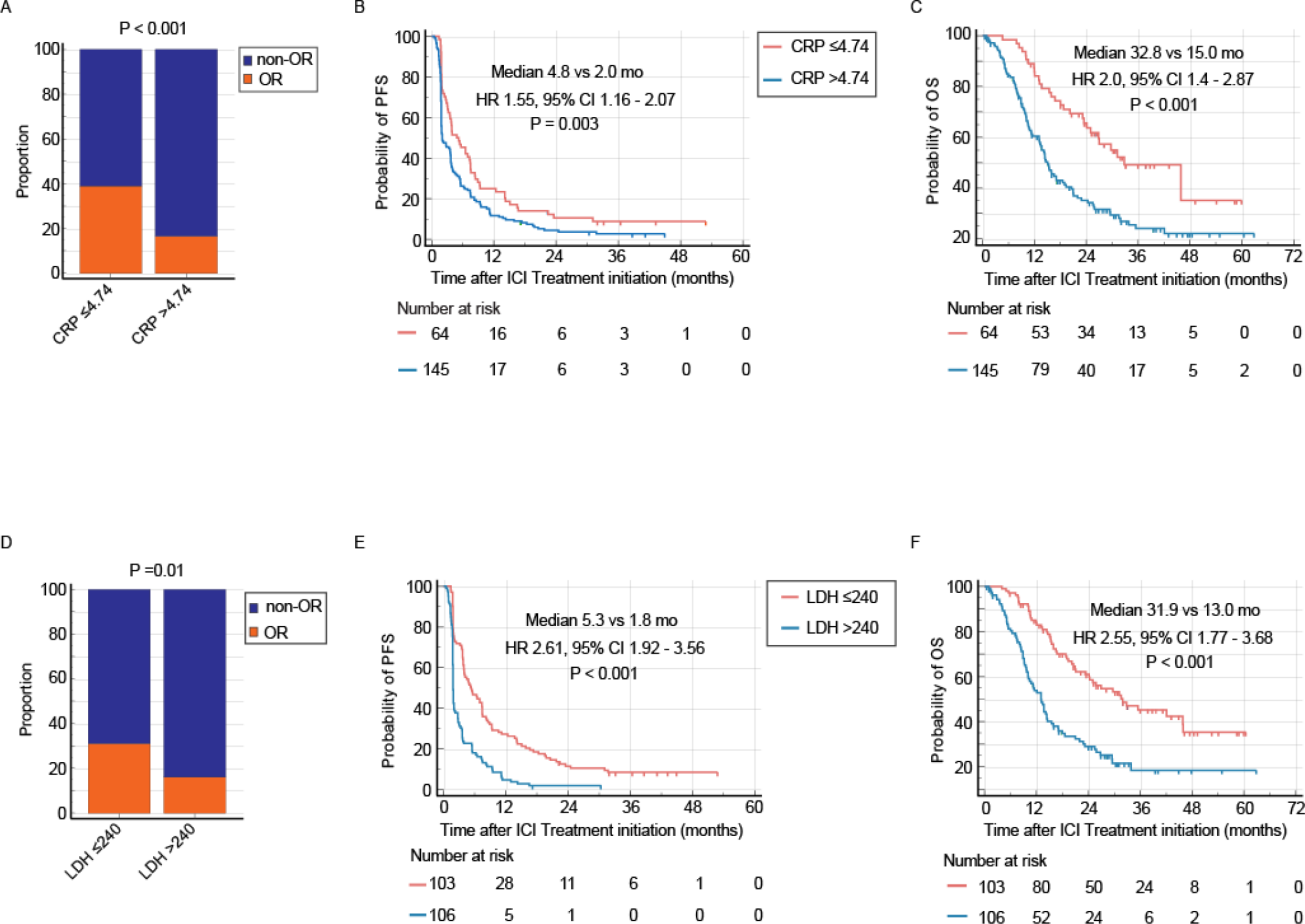
Prognostic and predictive value of pretreatment CRP and LDH levels. Elevated baseline levels of both C-reactive protein (CRP > 4.74 mg/L) and lactate dehydrogenase (LDH > 240 U/L) are associated with inferior clinical outcomes in patients with RM-NPC treated with PD-1 inhibitor monotherapy. **(A-C)** Comparison of objective response rate (ORR, **A**), progression-free survival (PFS, **B**), and overall survival (OS, **C**) between patients with low (≤4.74 mg/L) and high (>4.74 mg/L) pretreatment CRP. **(D-F)** Comparison of ORR **(D)**, PFS **(E)**, and OS **(F)** between patients with low (≤240 U/L) and high (>240 U/L) pretreatment LDH.

Elevated baseline levels of CRP and LDH were both significantly associated with inferior responses to PD-1 inhibitor monotherapy. Patients with low baseline CRP achieved an objective response rate (ORR) of 39.1%, compared to 16.6% in those with high baseline CRP (P < 0.001, **Figure 2A**). Similarly, a low baseline LDH (≤240 U/L) was associated with a higher ORR (31.1%) than a high baseline LDH (>240 U/L, ORR 16%, P = 0.01, **Figure 2D**).

Survival outcomes followed a consistent pattern. The low baseline CRP group demonstrated significantly longer median progression-free survival (mPFS: 4.8 vs. 2.3 months; HR 1.55, 95% CI 1.16–2.07, P = 0.003) and median overall survival (mOS: 32.8 vs. 15.1 months; HR 2.0, 95% CI 1.4–2.9, P < 0.001) compared to the high baseline CRP group (**Figures 2B, C**). Likewise, high baseline LDH was associated with shorter mOS (HR 2.55; 95% CI 1.77–3.68; P < 0.001) and mPFS (HR 2.61; 95% CI 1.92–3.56; P < 0.001, **Figures 2E, F**).

### Association of CRP and LDH kinetics with treatment outcomes

**Figure 3:**
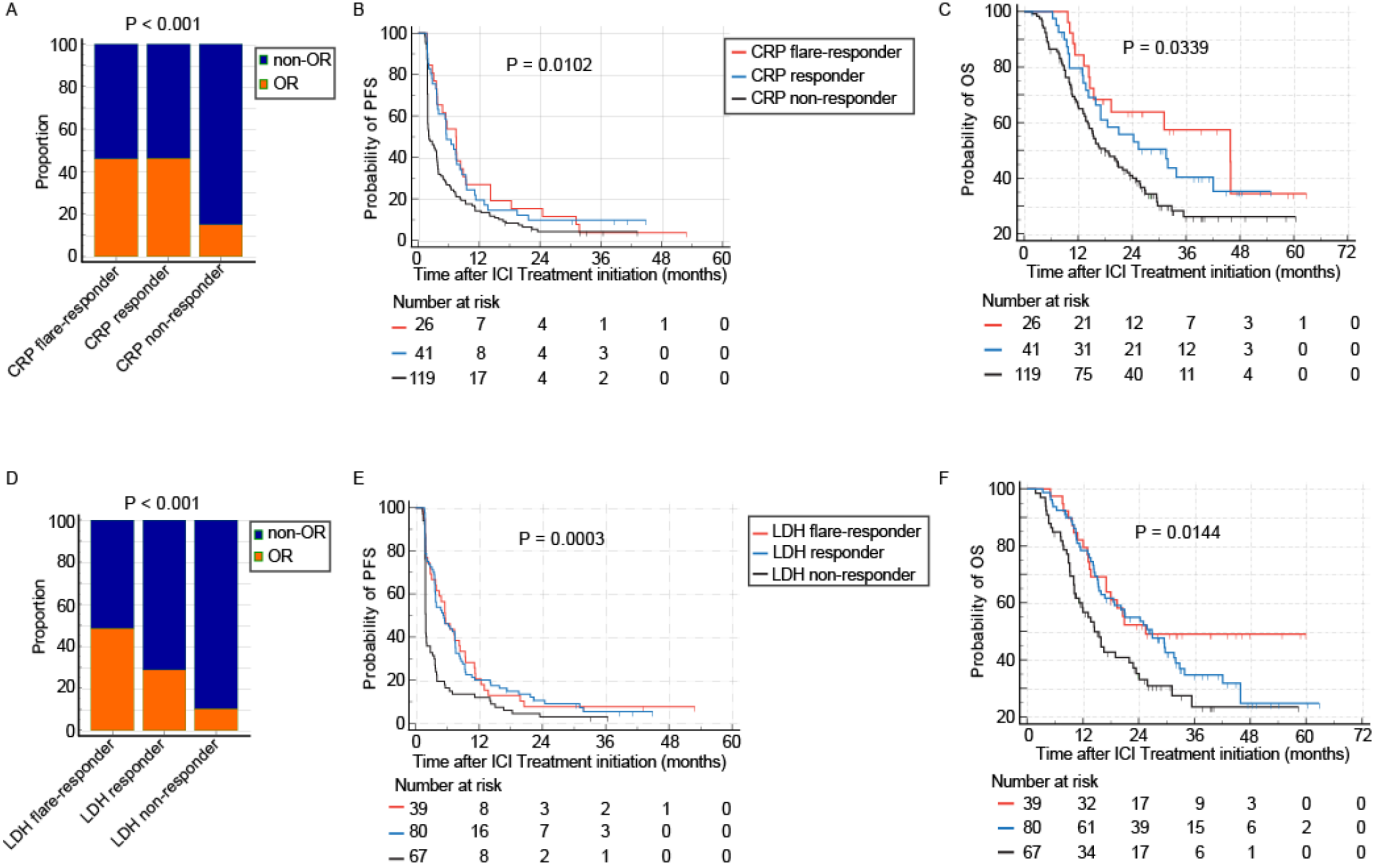
Association of early on-treatment biomarker kinetics with clinical outcomes. Dynamic changes in CRP and LDH following PD-1 inhibitor initiation serve as powerful predictors of therapeutic efficacy. **(A-C)** Outcomes based on CRP kinetics: Patients classified as CRP non-responders exhibited the lowest ORR **(A)**, shortest PFS **(B)**, and poorest OS **(C)** compared to intermediate and good responders. **(D-F)** Outcomes based on LDH kinetics: A similar pattern was observed for LDH non-responders, who demonstrated the lowest ORR **(D)**, shortest PFS **(E)**, and poorest OS **(F)**.

Dynamic changes in CRP and LDH during treatment provided further prognostic stratification. Based on predefined kinetic criteria, CRP responses were categorized as flare responders (n=26, 14%), responders (n=41, 22%), or non-responders (n=119, 64%). The ORR was 46.2% for flare responders, 46.3% for responders, and 15.1% for non-responders (P < 0.001, **Figure 3A**). A similar gradient was observed in survival outcomes: mPFS was 7.4 months (95% CI, 3.7–8.9), 5.5 months (95% CI, 3.6–8.2), and 2.1 months (95% CI, 1.9–3.6), respectively (P = 0.0102, **Figure 3B**); mOS was 45.8 months (95% CI, 15.4–45.9), 31.5 months (95% CI, 15.9–42.0), and 18.0 months (95% CI, 14.3–23.4), respectively (P = 0.0339, **Figure 3C**).

Similarly, LDH kinetics identified three groups: flare responders (n=39, 21%), responders (n=80, 43%), and non-responders (n=67, 36%). Their respective ORRs were 48.7%, 28.7%, and 10.4% (P < 0.001, **Figure 3D**). The mPFS for these groups was 1.9 months (95% CI, 1.8–2.0), 5.2 months (95% CI, 3.6–7.4), and 5.5 months (95% CI, 3.0–8.4, **Figure 3E**); mOS was 14.5 months (95% CI, 10.5–22.8), 26.8 months (95% CI, 16.7–32.8), and 25.5 months (95% CI, 17.0–25.5, **Figure 3F**).

Given the comparable ORR between flare responders and responders for both biomarkers, these two categories were combined into a single “response group” for subsequent analyses (**Supplementary Figure 2**).

### Pre-treatment immune response index

**Figure 4:**
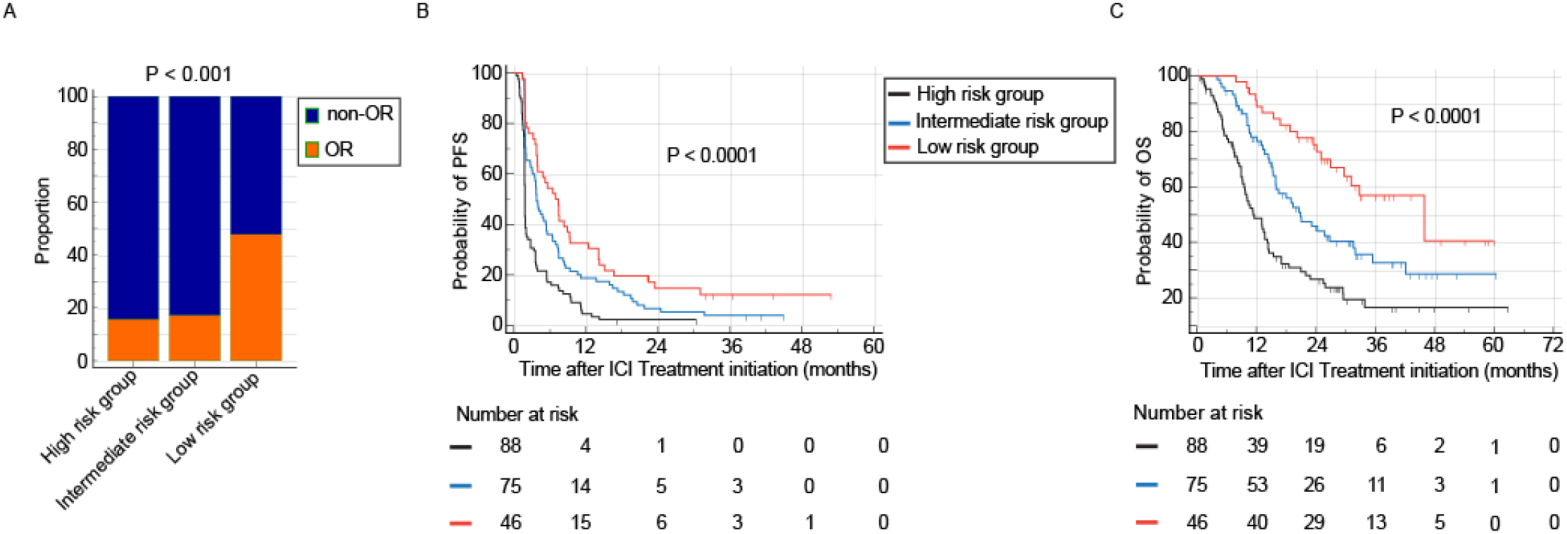
Prognostic stratification using the pretreatment Immune Response Index (IRI). Patients classified into the high-risk group by the pretreatment IRI exhibited significantly inferior clinical outcomes compared to low- and intermediate-risk groups. **(A-C)** Comparative analysis of objective response rate (ORR, **A**), progression-free survival (PFS, **B**), and overall survival (OS, **C**) across the three pretreatment IRI risk strata (Low, Intermediate, High). The high-risk group demonstrated the lowest ORR, the shortest median PFS, and the shortest median OS.

Given that all candidate variables demonstrated minimal multicollinearity with variance inflation factors (VIF) less than 3 (maximum VIF: 1.305), all were included in the Cox regression model. The subsequent multivariable analyses identified baseline CRP and LDH as independent predictors of overall survival (**Table 2**; **Supplementary Table 2**). Consequently, these two biomarkers were integrated to construct a pre-treatment Immune Response Index (IRI) for stratifying patients prior to receiving PD-1 inhibitor monotherapy. Among the 209 patients, 46 (22.0%) were classified as low risk, 75 (35.9%) as intermediate risk, and 88 (42.1%) as high risk. Objective response rates (ORR) were 47.8% in the low-risk group, 17.3% in the intermediate-risk group, and 15.9% in the high-risk group (P = 0.0001, **Figure 4A**). By the time of analysis, a total of 198 PFS events and 126 OS events had been recorded in the entire cohort. Correspondingly, the absolute number of PFS and OS events within each subgroup are detailed in **Supplementary Table 3**. The median PFS was 6.9 months (95% CI, 3.9–9.3), 3.8 months (95% CI, 3.0–5.4), and 1.8 months (95% CI, 1.8–1.9) in the low-, intermediate-, and high-risk groups, respectively (P < 0.0001, **Figure 4B**). Similarly, the median OS was 45.9 months (95% CI, 29.7–45.9), 20.9 months (95% CI, 15.9–31.9), and 11.3 months (95% CI, 9.2–14.3), respectively (P < 0.0001, **Figure 4C**). To assess the independent prognostic value of the pre-treatment IRI, a separate multivariable Cox model was constructed. Given that IRI is derived from CRP and LDH, these two biomarkers were excluded from the model to avoid structural multicollinearity. The analysis adjusted for all other clinical covariates and demonstrated that the IRI remained an independent predictor of both OS and PFS (**Supplementary Tables 4**, **5**). To provide a quantitative tool for individualized risk assessment, nomograms integrating pre-treatment IRI with other clinical factors were constructed for OS and PFS, respectively (**Supplementary Figures 3A, C**). Decision curve analysis showed that the IRI−incorporated nomogram provided superior net clinical benefit across a wide range of threshold probabilities compared to alternative models (**Supplementary Figures 3B, D**). These results indicate that the pre-treatment IRI is associated with patient prognosis and may serve as a factor for risk stratification prior to PD-1 inhibitor therapy.

**Table 2:** Univariable and multivariable analyses for OS.

Characteristics	Univariable		Multivariable	
	Hazard ratio (95% CI)	P value	Hazard ratio (95% CI)	P value
Age, years				
≤45	Reference			
>45	1.07 (0.75,1.53)	0.696		
Sex				
Male	Reference			
Female	1.19 (0.74,1.90)	0.476		
Drinking				
No	Reference			
Yes	1.42 (0.78,2.57)	0.254		
Smoking				
No	Reference			
Yes	1.21 (0.83,1.75)	0.325		
Body mass index, kg/m2				
<18.5	Reference			
18.5-23.9	0.72 (0.45,1.153)	0.146		
24.0-27.9	0.61 (0.32,1.14)	0.122		
≥28.0	1.18 (0.35,3.93)	0.793		
EOCG				
0	Reference			
1	1.41 (0.96,2.07)	0.081		
Disease type				
Metastasis	Reference			
Recurrence	0.31 (0.11,0.87)	0.021		
Both	0.77 (0.46,1.28)	0.307		
No of metastatic sites				
0	Reference			
1-3	1.97 (0.53,7.33)	0.313		
≥4	3.20 (1.18,8.66)	0.022		
ALB, g/L				
≤44	Reference		Reference	
>44	0.62 (0.43,0.89)	0.01	0.64 (0.45,0.93)	0.018
CRP, mg/L				
≤4.74	Reference		Reference	
>4.74	2.18 (1.44,3.29)	0	1.58 (1.03,2.42)	0.038
LDH, U/L				
≤240	Reference		Reference	
>240	2.46 (1.72,3.53)	0	2.14 (1.47,3.12)	0
NLR				
≤3.7	Reference			
>3.7	1.0 (0.02,67.5)	1		
EBV-DNA, copy/ml				
≤1490	Reference		Reference	
>1490	2.04 (1.27,3.30)	0.003	1.72 (1.07,2.79)	0.027

CI, Confidence interval; ECOG, Eastern Cooperative Oncology Group Performance Status; ALB, Albumin; CRP, C-reaction protein; LDH, Lactate dehydrogenase; HGB, Hemoglobin; NLR, Neutrophil-to-lymphocyte ratio; EBV-DNA, Epstein-Barr virus DNA. Hazard ratios were estimated by Cox proportional hazards regression.

### On-treatment immune response index

**Figure 5:**
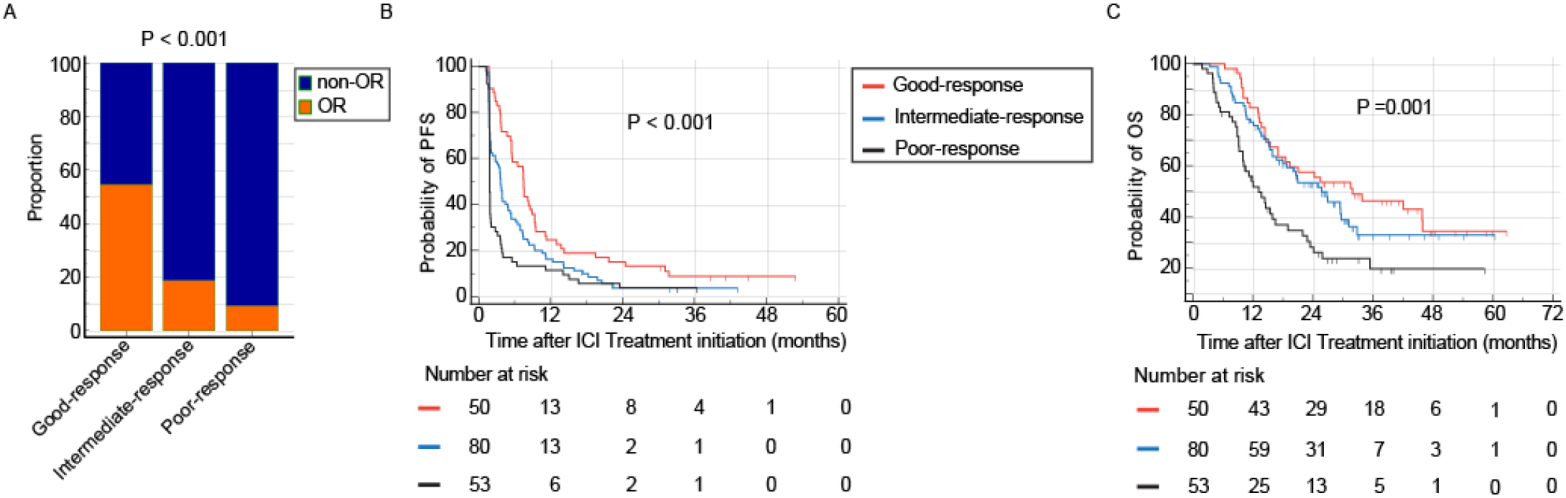
Dynamic risk stratification by the on-treatment Immune Response Index (IRI). Early on-treatment assessment using the IRI effectively identifies patients with poor therapeutic outcomes following PD-1 inhibitor monotherapy. **(A-C)** Clinical outcomes across on-treatment IRI response groups (Good, Intermediate, Poor). The poor-response group demonstrated the lowest objective response rate (ORR, **A**), shortest progression-free survival (PFS, **B**), and shortest overall survival (OS, **C**).

Although both CRP and LDH response were associated with improved immunotherapy outcomes, approximately 15% of patients in the non-response groups for each biomarker still achieved an objective response. To more accurately identify patients unlikely to benefit from extended immunotherapy, we integrated the kinetic patterns of CRP and LDH to develop an on-treatment IRI. A total of 209 patients with recurrent or metastatic nasopharyngeal carcinoma who received anti-PD-1 therapy were enrolled. Of these, 23 patients (11.0%) were excluded due to very early disease progression occurring before the first on-treatment assessment or insufficient follow-up for kinetic classification. The remaining 186 patients were stratified into three dynamic response subgroups based on the composite on-treatment IRI. therefore, the on-treatment IRI is applicable for dynamic risk reassessment after early treatment exposure, rather than for baseline prediction. Among the 186 patients included in the longitudinal analysis, 53 (28.5%) were categorized into the good-response group, 80 (43.0%) into the intermediate-response group, and 53 (28.5%) into the poor-response group. Objective response rates (ORR) were 54.7% in the good-response group, 18.8% in the intermediate-response group, and 9.4% in the poor-response group (P < 0.001, **Figure 5A**). The good-response group demonstrated the longest median PFS of 7.5 months (95% CI: 5.5–8.9), compared to 3.7 months (95% CI: 2.3–4.8) in the intermediate-response group and 1.8 months (95% CI: 1.8–1.9) in the poor-response group. Similarly, median OS was 31.9 months (95% CI: 17.0–45.9), 25.7 months (95% CI: 18.0–31.1), and 13.1 months (95% CI: 10.0–19.0) across the three groups, respectively (both P ≤ 0.001, **Figures 5B, C**). The absolute number of PFS and OS events recorded within each of these on-treatment subgroups is detailed in **Supplementary Table 6**. These results suggest that the on-treatment IRI contributes to the early stratification of treatment outcomes, providing a basis for future studies to investigate its utility in monitoring and evaluating immunotherapy duration, subject to prospective validation.

### On-treatment IRI stratified by baseline EBV DNA subgroups

Given that baseline plasma EBV DNA level is a known prognostic factor in advanced nasopharyngeal carcinoma (NPC), we further evaluated the performance of the on-treatment IRI within patient subgroups stratified by baseline EBV DNA status. The cohort was divided into high and low baseline EBV DNA subgroups to assess whether the predictive utility of the on-treatment IRI varied according to baseline viral load. In the high baseline EBV DNA subgroup, the on-treatment IRI demonstrated strong discriminatory ability. Significant differences were observed in ORR, with rates of 46.7% in the good-response group, 14.3% in the intermediate-response group, and 0% in the poor-response group (P < 0.001). Similarly, progressive stratification was seen in survival outcomes: median PFS was 7.5 (95% CI: 5.5–8.9), 3.1 (95% CI: 1.9–3.9), and 1.8 (95% CI: 1.8–1.9) months, and median OS was 31.9 (95% CI: 14.6–45.9), 25.0 (95% CI: 14.2–31.1), and 11.2 (95% CI: 8.9–15.4) months across the three response groups, respectively (P < 0.001; **Supplementary Figures 4A–C**). These results indicate that the on-treatment IRI shows enhanced prognostic association in patients with high baseline EBV DNA, highlighting its role as a candidate tool for risk-stratified monitoring in this subgroup, which merits further investigation.

In contrast, within the low baseline EBV DNA subgroup, the on-treatment IRI did not show statistically significant differential trends in ORR, PFS, or OS among the response groups (**Supplementary Figures 4D–F**). It is important to note that this analysis was exploratory and limited by the small sample size of this subset, and the findings should be interpreted with caution.

## Discussion

This study introduces and validates an integrated immune response index (IRI) based on the systemic inflammatory markers C-reactive protein (CRP) and lactate dehydrogenase (LDH). The index incorporates both pretreatment levels and on-treatment kinetic changes, offering a potential tool for prognostic stratification and response monitoring in patients with recurrent or metastatic nasopharyngeal carcinoma (RM-NPC) undergoing PD-1 inhibitor monotherapy. This composite model may offer advantages over single-marker or static baseline assessments in monitoring response patterns, supporting its further evaluation as a tool for prognostic monitoring and dynamic risk stratification.

Consistent with evidence from other malignancies, our results substantiate a significant inverse relationship between pre-treatment systemic inflammatory status and clinical outcomes following PD-1 blockade. Elevated baseline levels of CRP and LDH were strongly correlated not only with reduced objective response rates but also with shorter progression-free and overall survival in our cohort. These findings align with prior reports in melanoma and non-small cell lung cancer, where markers of systemic inflammation have been consistently associated with inferior responses to immune checkpoint inhibitors (ICI) (14, 17). The biological rationale underlying this correlation is well-established. Chronic systemic inflammation can promote the development of an immunosuppressive tumor microenvironment. This occurs through mechanisms such as the recruitment and activation of immunosuppressive cells, including myeloid-derived suppressor cells and regulatory T cells, the upregulation of inhibitory immune checkpoint molecules, and the induction of metabolic shifts that support tumor growth and evasion (18). In the context of EBV-associated NPC, this systemic inflammation may be further amplified by viral latency proteins and lytic reactivation, which can directly modulate inflammatory pathways and immune cell function (15, 19). Thus, the pre-treatment IRI likely captures not only a generic immunosuppressive state but also the magnitude of this virus-host-inflammatory interplay, explaining its particular stratification power in this disease. Thus, in recurrent or metastatic nasopharyngeal carcinoma treated with PD-1 monotherapy, the pre-treatment IRI identifies a patient subset associated with poorer response. This finding supports further evaluation of the index as a potential stratification tool prior to treatment initiation.

The integration of dynamic biomarker kinetics represents a key methodological advance in our study. We observed that the on-treatment IRI may have improved risk-stratification capability compared to early changes in CRP or LDH alone. Patients categorized within the “good-response” group by the on-treatment IRI achieved a median PFS of 7.5 months and a median OS of 31.9 months, with an ORR surpassing 50%. These data imply that a rapid decline in systemic inflammation following treatment initiation may serve as an early pharmacodynamic signal, reflecting successful immune engagement and a shift towards a more immunopermissive tumor microenvironment. Notably, approximately 15% of patients whose individual biomarkers were classified as “non-responders” still attained an objective response, underscoring the limitation of unidimensional kinetic assessment. In contrast, the composite on-treatment IRI effectively identified a subset of patients with recurrent or metastatic nasopharyngeal carcinoma who were likely to have a poor response to PD-1 monotherapy, as evidenced by a substantially lower objective response rate (ORR) of 9.4%. This finding carries clinical relevance. For patients stratified into this “poor-response” group by the on-treatment IRI, a more vigilant clinical monitoring approach may be warranted to optimize clinical management. For patients with suboptimal responses to PD-1 inhibitors, combination strategies represent a promising therapeutic direction. Accumulating evidence supports the synergistic effect of immunotherapy and anti-angiogenic agents (20, 21). This synergy is exemplified by a Phase II trial of famitinib plus camrelizumab in patients with recurrent/metastatic NPC who had failed prior PD-1 blockade, which reported an objective response rate of 33.3% and a median progression-free survival of 7.2 months (22). This provides an evidence-based subsequent-line option for the very patient subgroup that our on-treatment IRI may help identify early.

Another key finding was the heterogeneity in the predictive performance of the on-treatment IRI across patient subgroups. It demonstrated excellent risk-stratification capability within the subgroup of patients with high baseline EBV DNA load, showing clear separation in survival curves among response groups. A plausible explanation is that a high EBV DNA load is often associated with greater tumor burden, more pronounced virus-related inflammation, and a poorer prognosis (23, 24). In these patients, the efficacy of PD-1 inhibitors may vary more substantially, and the on-treatment IRI appears sensitive in capturing the differential treatment effects reflected by inflammatory dynamics. These findings thus support the prospective evaluation of the on-treatment IRI as a tool for risk-stratified and response monitoring in patients with elevated tumor burden or systemic inflammation. Conversely, in patients with a lower baseline risk (low EBV DNA), the overall prognosis is relatively better, and treatment response may be influenced more by other factors (e.g., tumor mutational burden, host genomic features) (25, 26), potentially diluting the predictive signal from inflammatory kinetics. Our exploratory subgroup analysis aligns with this notion, showing an attenuated prognostic value of the on-treatment IRI among patients with low baseline EBV DNA. However, given the limited sample size and consequent low statistical power, this finding requires validation in larger, independent cohorts. Hence, the on-treatment IRI merits prospective validation in larger cohorts to evaluate its role in risk-stratified monitoring across patients with varying levels of tumor burden.

### Limitation

We acknowledge several limitations of this study. First, its single-center, retrospective nature necessitates validation in multicenter, prospective cohorts. Second, optimal cut-offs for biomarkers such as CRP (4.74 mg/L) and LDH (240 U/L) were determined by ROC analysis within this cohort. This data-driven approach is susceptible to overfitting and may limit generalizability. Accordingly, external validation in independent cohorts and across assay platforms is required prior to broader clinical application. Third, the on-treatment IRI analysis is subject to guarantee-time bias, as 23 patients (11.0%) with very early progression were excluded from kinetic classification. This exclusion may have enriched the cohort for patients with more favorable short-term outcomes, potentially overestimating the prognostic value of on-treatment IRI. Fourth, while no severe multicollinearity was detected via VIF analysis, the biological correlation between inflammatory markers and tumor burden indicators (e.g., EBV DNA) means residual confounding remains possible. This may affect the robustness of the estimated independent effect of IRI. Finally, while we included EBV DNA in our analysis, other potential biomarkers such as PD-L1 expression and tumor-infiltrating lymphocytes were not integrated. Future research should aim to combine the IRI with these and other multidimensional biomarkers to build more comprehensive predictive models.

## Conclusion

In conclusion, the IRI—based on pretreatment levels and on-treatment kinetics of CRP and LDH—offers a practical framework for prognostic stratification in patients with recurrent or metastatic nasopharyngeal carcinoma receiving PD-1 inhibitor monotherapy. It provides a structured approach to baseline risk assessment and dynamic response monitoring. These findings support the continued evaluation of the IRI as a prognostic biomarker in NPC immunotherapy. Future studies are warranted to prospectively validate its performance and to explore integration with other clinical or biological variables.

## Data Availability

The datasets presented in this article are not readily available because The datasets generated and/or analyzed during the current study are not publicly available due to patient privacy and confidentiality concerns, as stipulated by the ethical approval and institutional policies. However, de-identified data supporting the key findings of this study are available from the corresponding author upon reasonable request. Data requests will be reviewed by the institutional ethics committee to ensure compliance with ethical guidelines. Access may be granted for legitimate scientific research purposes following the execution of a data use agreement. Requests to access the datasets should be directed to chenjiamin1005@163.com.
