# Supplementary material for: A dynamic immune response index combining C-reactive protein and lactate dehydrogenase kinetics is associated with outcomes of PD-1 inhibitor monotherapy in recurrent/metastatic nasopharyngeal carcinoma
Source: Front Oncol. 2026 Mar 13;16:1775584. doi: 10.3389/fonc.2026.1775584 (PMC13021470; doi:10.3389/fonc.2026.1775584)
Supplement: Supplementary file 1 [file DataSheet1.pdf]

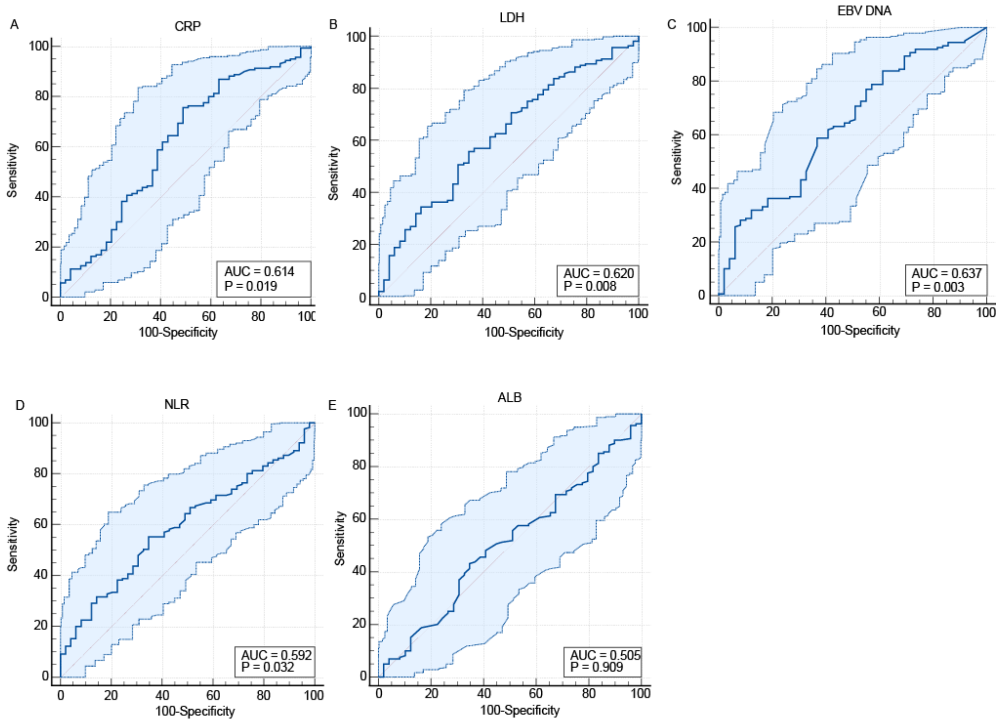

Fig. S1. Determination of optimal cutoff values for baseline biomarkers. Receiver operating characteristic (ROC) curve analysis was performed to identify the optimal pretreatment cutoff levels of serum biomarkers for predicting primary resistance (progressive disease as the endpoint) in the cohort of RM-NPC patients receiving PD-1 inhibitor monotherapy. The Youden index was used to determine the optimal threshold. Panels display the ROC curves for (A) C-reactive protein (CRP), (B) lactate dehydrogenase (LDH), (C) plasma Epstein-Barr virus (EBV) DNA, (D) neutrophil-to-lymphocyte ratio (NLR), and (E) albumin (ALB).

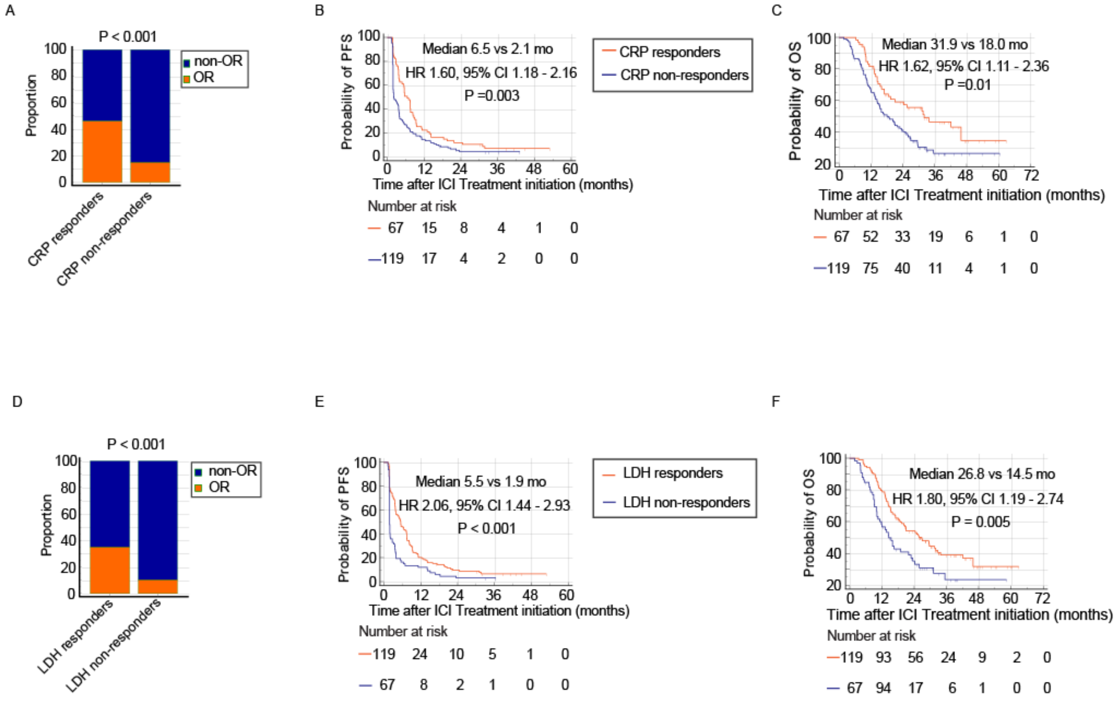

Fig. S2. Prognostic impact of biomarker non-response defined by individual CRP or LDH kinetics. Patients were stratified into responders and non-responders based on the lack of a significant early decrease in either C-reactive protein (CRP) or lactate dehydrogenase (LDH) levels following treatment initiation. (A-C) Comparison of objective response rate (ORR, A), progression-free survival (PFS, B), and overall survival (OS, C) between CRP responders and CRP non-responders. The non-response group exhibited inferior outcomes across all endpoints. (D-F) Comparison of ORR (D), PFS (E), and OS (F) between LDH responders and LDH non-responders, demonstrating a consistent pattern of poorer outcomes in the non-response group.

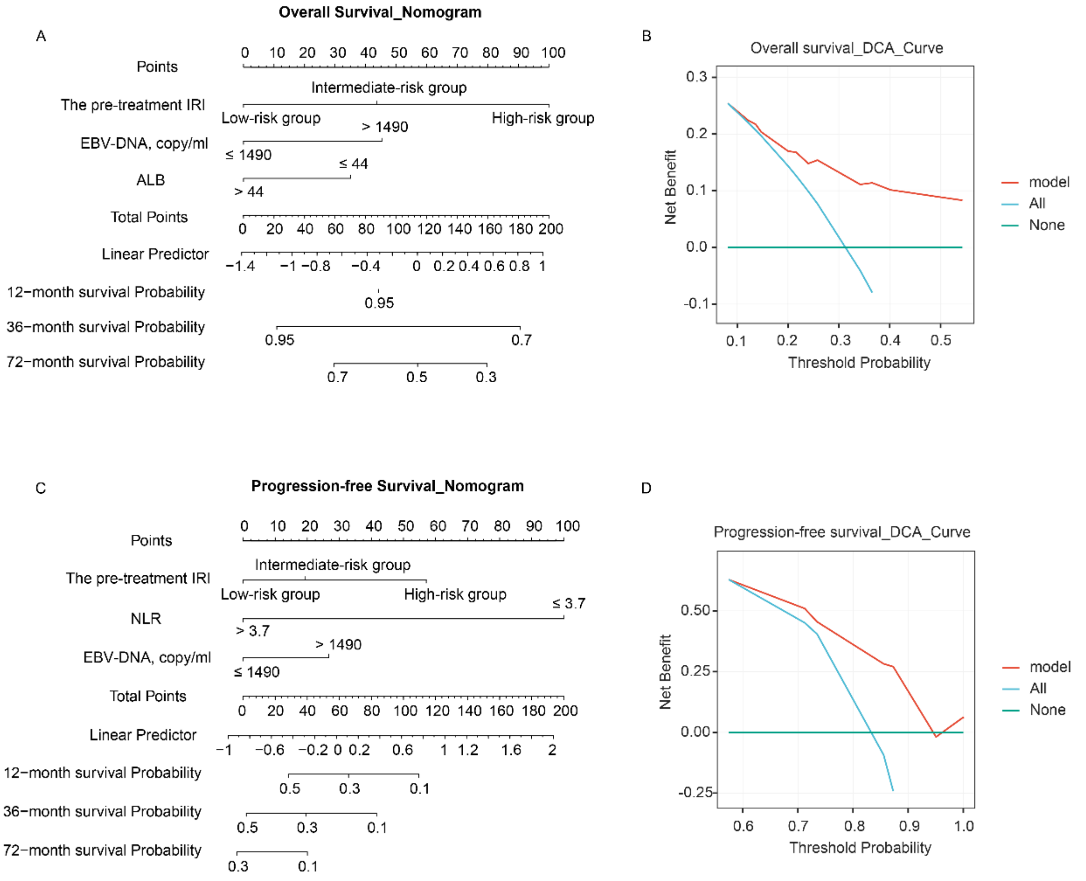

Fig. S3. Prognostic value of the pre-treatment Inflammatory Response Index (IRI) in survival prediction. (A) Nomogram incorporating pre-treatment IRI and key clinical variables for predicting 12-, 36-, and 72-month overall survival (OS). (B) Decision curve analysis (DCA) of the nomogram for OS prediction, demonstrating its clinical net benefit. (C) Nomogram integrating pre-treatment IRI for predicting 12-, 36-, and 72-month progression-free survival (PFS). (D) DCA of the PFS prediction nomogram.

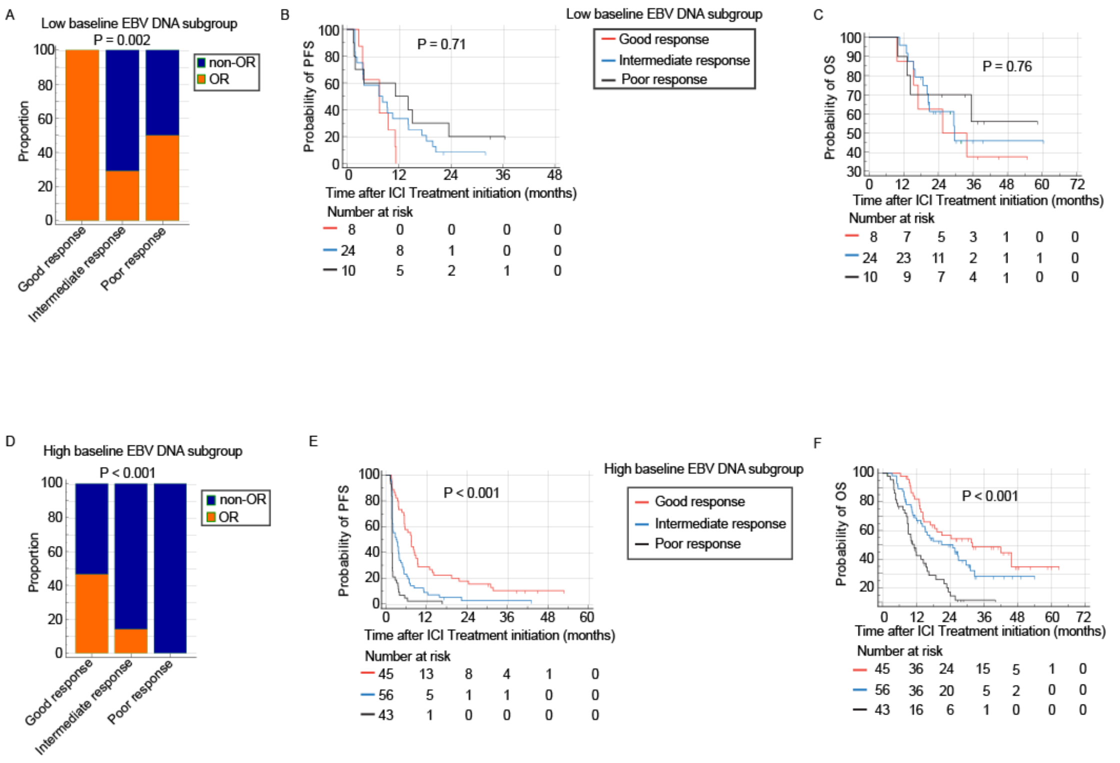

Fig. S4. Prognostic performance of the on-treatment IRI across patient subgroups stratified by baseline plasma EBV DNA load. The predictive utility of the on-treatment Immune Response Index (IRI) was evaluated in patients with low (≤1490 copies/mL) and high (>1490 copies/mL) baseline EBV DNA levels. (A-C) In the low EBV DNA subgroup, the good-response group achieved a 100% objective response rate (ORR, A). However, no statistically significant differences in progression-free survival (PFS, B) or overall survival (OS, C) were observed among the three IRI response groups. (D-F) Conversely, in the high EBV DNA subgroup, the on-treatment IRI demonstrated robust risk stratification: the good-response group showed the highest ORR (D), along with significantly longer PFS (E) and OS (F) compared to intermediate- and poor-response groups.

Table.S1 Measurement method and reference value of parameters

Test item	Instrument	Medical reference value	Unit
ALB	LST008AS	40~55	g/L
CRP	LST008AS	0-3.0	mg/L
LDH	LST008AS	120~250	U/L
Neutrophil	XN9000	1.8~6.3	10E9/L
Lymphocyte	XN9000	1.1~3.2	10E9/L

Abbreviations: ALB=Albumin; CRP=C-reaction protein; LDH=Lactate dehydrogenase.

Table.S2 Univariable and multivariable analyses for PFS.

Characteristics	Univariable Hazard ratio (95% CI)	Univariable P value	Multivariable Hazard ratio (95% CI)	Multivariable P value
Age, years				
≤45	Reference			
>45	0.84 (0.63,1.11)	0.209		
Sex				
Male	Reference			
Female	0.81 (0.55,1.20)	0.298		
Drinking				
No	Reference			
Yes	1.72 (1.03,2.88)	0.039	1.50 (0.89,2.52)	0.128
Smoking				
No	Reference			
Yes	1.18 (0.87,1.60)	0.277		
Body mass index, kg/m2				
<18.5	Reference			
18.5-23.9	1.0 (0.68,1.47)	0.98		
24.0-27.9	0.94 (0.57,1.54)	0.793		
≥28.0	1.67 (0.59,4.76)	0.338		
EOCG				
0	Reference			
1	1.11 (0.82,1.51)	0.482		
Disease type				
Metastasis	Reference			
Recurrence	0.48 (0.27,0.85)	0.011		
Both	0.71 (0.48,1.06)	0.091		
Recurrence type				
None	Reference			
local recurrence	0.74 (0.33,1.68)	0.476		
Regional recurrence	0.55 (0.35,0.88)	0.012		
Both	0.76 (0.44,1.31)	0.315		
No of metastatic sites				
0	Reference			
1-3	1.58 (0.67,3.70)	0.293		
≥4	1.98 (1.12,3.50)	0.018		
ALB, g/L				
≤44	Reference			
>44	0.87 (0.66,1.16)	0.339		
CRP, mg/L				
≤4.74	Reference		Reference	
>4.74	1.59 (1.17,2.16)	0.003	1.15 (0.83,1.59)	0.403

LDH, U/L				
≤240	Reference		Reference	
>240	2.41 (1.8,3.23)	0	1.97 (1.44,2.70)	0
NLR				
≤3.7	Reference			
>3.7	0.14 (0.08,0.26)	0	0.22 (0.12,0.41)	0
EBV-DNA, copy/ml				
≤1490	Reference		Reference	
>1490	1.79 (1.27,2.54)	0.001	1.50 (1.05,2.14)	0.027

Abbreviations: CI=Confidence interval; ECOG=Eastern Cooperative Oncology Group Performance Status; ALB=Albumin; CRP=C-reaction protein; LDH=Lactate dehydrogenase; NLR =Neutrophil-to-lymphocyte ratio; EBV-DNA= Epstein-Barr virus DNA. Hazard ratios were estimated by Cox proportional hazards regression.

Table.S3 Absolute Number of PFS and OS Events Across Pre-treatment IRI Subgroups

Subgroup	Total Patients	Patients with PFS Event	Patients with OS Event
Low-risk group	46	40 (86.96%)	19 (41.30%)
Intermediate-risk group	75	72 (96.00%)	43 (57.33%)
High-risk group	88	86 (97.73%)	64 (72.73%)
Overall	209	198 (94.74%)	126 (60.29%)

Abbreviations: PFS= Progression-Free Survival; OS= Overall Survival; IRI= Immune Response Index.

Table.S4 Univariable and multivariable analyses for OS.

Characteristics	Univariable		Multivariable	
	Hazard ratio (95% CI)	P value	Hazard ratio (95% CI)	P value
Age, years				
≤45	Reference			
>45	1.07 (0.75,1.53)	0.696		
Sex				
Male	Reference			
Female	1.19 (0.74,1.90)	0.476		
Drinking				
No	Reference			

Yes	1.42 (0.78,2.57)	0.254		
Smoking				
No	Reference			
Yes	1.21 (0.83,1.75)	0.325		
Body mass index, kg/m2				
<18.5	Reference			
18.5-23.9	0.72 (0.45,1.153)	0.146		
24.0-27.9	0.61 (0.32,1.14)	0.122		
≥28.0	1.18 (0.35,3.93)	0.793		
EOCG				
0	Reference			
1	1.41 (0.96,2.07)	0.081		
Disease type				
Metastasis	Reference			
Recurrence	0.31 (0.11,0.87)	0.021		
Both	0.77 (0.46,1.28)	0.307		
Pre-treatment IRI				
The low-risk group	Reference		Reference	
The intermediate-risk group	1.93 (1.12,3.32)	0.017	1.70 (0.98,2.93)	0.057
The high-risk group	3.74 (2.23,6.27)	0	3.37 (2.00,5.68)	0
ALB, g/L				
≤44	Reference		Reference	
>44	0.62 (0.43,0.89)	0.01	0.65 (0.45,0.94)	0.021
NLR				
≤3.7	Reference			
>3.7	1.0 (0.02,67.5)	1		
EBV-DNA, copy/ml				
≤1490	Reference		Reference	
>1490	2.04 (1.27,3.30)	0.003	1.73 (1.07,2.80)	0.025

Abbreviations: CI=Confidence interval; ECOG=Eastern Cooperative Oncology Group Performance Status; ALB=Albumin; CRP=C-reaction protein; LDH=Lactate dehydrogenase; HGB=Hemoglobin; NLR =Neutrophil-to-lymphocyte ratio; EBV-DNA= Epstein-Barr virus DNA; IRI= Immune Response Index. Hazard ratios were estimated by Cox proportional hazards regression.

Table.S5 Univariable and multivariable analyses for PFS.

Characteristics	**Univariable**		**Multivariable**	
	Hazard ratio (95% CI)	P value	Hazard ratio (95% CI)	P value
Age, years				
≤45	Reference			
>45	0.84 (0.63,1.11)	0.209		
Sex				

Male	Reference			
Female	0.81 (0.55,1.20)	0.298		
Drinking				
No	Reference			
Yes	1.72 (1.03,2.88)	0.039	1.53 (0.90,2.58)	0.114
Smoking				
No	Reference			
Yes	1.18 (0.87,1.60)	0.277		
Body mass index, kg/m2				
<18.5	Reference			
18.5-23.9	1.0 (0.68,1.47)	0.98		
24.0-27.9	0.94 (0.57,1.54)	0.793		
≥28.0	1.67 (0.59,4.76)	0.338		
EOCG				
0	Reference			
1	1.11 (0.82,1.51)	0.482		
Disease type				
Metastasis	Reference		Reference	
Recurrence	0.48 (0.27,0.85)	0.011	0.65 (0.36,1.19)	0.16
Both	0.71 (0.48,1.06)	0.091	0.85 (0.56,1.30)	0.446
Pre-treatment IRI				
The low-risk group	Reference		Reference	
The intermediate-risk group	1.45 (0.98,2.14)	0.06	1.44 (0.96,2.17)	0.08
The high-risk group	2.86 (1.95,4.21)	0	2.18 (1.45,3.28)	0
No of metastatic sites				
0	Reference			
1-3	1.58 (0.67,3.70)	0.293		
≥4	1.98 (1.12,3.50)	0.018		
ALB, g/L				
≤44	Reference			
>44	0.87 (0.66,1.16)	0.339		
NLR				
≤3.7	Reference			
>3.7	0.14 (0.08,0.26)	0	0.22 (0.12,0.41)	0
EBV-DNA, copy/ml				
≤1490	Reference		Reference	
>1490	1.79 (1.27,2.54)	0.001	1.43 (0.99,2.05)	0.057

Abbreviations: CI=Confidence interval; ECOG=Eastern Cooperative Oncology Group Performance Status; ALB=Albumin; CRP=C-reaction protein; LDH=Lactate dehydrogenase; NLR =Neutrophil-to-lymphocyte ratio; EBV-DNA= Epstein-Barr virus DNA; IRI= Immune Response Index. Hazard ratios were estimated by Cox proportional hazards regression.

Table.S6 Absolute Number of PFS and OS Events Across On-treatment IRI Subgroups

Subgroup	Total Patients	Patients with PFS Event	Patients with OS Event
Good-response group	53	48 (90.57%)	30 (56.60%)
Intermediate-response group	80	76 (95.00%)	44 (55.00%)
Non-response group	53	51 (96.23%)	39 (73.58%)
Overall	186	175 (94.09%)	113 (60.75%)

Abbreviations: PFS= Progression-Free Survival; OS= Overall Survival; IRI= Immune Response Index.
